# Emotions and motivations of gambling: A comparison between scratch card, slot‐machines, and casino gamblers

**DOI:** 10.1002/brb3.3416

**Published:** 2024-03-07

**Authors:** Laura Ferro, Maria Grazia Monaci, Luca Scacchi

**Affiliations:** ^1^ Department of Social and Human Sciences (SHS) University of Aosta Valley Aosta Italy

**Keywords:** emotions, gambling, motivations

## Abstract

**Introduction:**

In the last 20 years, gambling has become more and more widespread in Italy. The aim of the present study is to compare the motivations for gambling and the emotions felt while gambling in three different subgroups: scratch card gamblers, slot‐machine gamblers, and casino gamblers.

**Methods:**

Three versions of a questionnaire have been distributed in a casino, in scratch card vendors, and slot‐machines venues. All versions included sociodemographic variables, the two‐question Lie‐Bet instrument, a scale for motivations toward gambling, and a list of positive and negative emotions felt while gambling.

**Results:**

Participants (*N* = 425, F 47.5%) are gamblers potentially pathological (*N* = 162, 38.1%) and not (*N* = 263, 61.9%). Different games seem connected to different motivations and emotions: the scratch gamblers show less Coping and Social Motivation and experience less Negative Emotion. However, the motivation and emotion most intensely experienced by the gamblers (Enhancement and the Positive Emotions) do not show differences between the different types of games.

**Conclusion:**

The pathological gamblers have a more intense and internal connection with the game (have more Enhancement and Coping motives, Positive and Negative Emotion). In sum, our findings support the theoretical hypothesis that gambling can be a tool for regulating emotions.

## Graphical abstract

The aim of the present study is to compare the motivations for gambling and the emotions felt while gambling in three different subgroups: scratch card gamblers, slot‐machine gamblers, and casino gamblers. Different games seem connected to different motivations and emotions: the scratch gamblers show less Coping and Social Motivation and experience less Negative Emotion. However, the motivation and emotion most intensely experienced by the gamblers (Enhancement and the Positive Emotions) do not show differences between the different types of games. Our findings support the theoretical hypothesis that gambling can be a tool for regulating emotions.

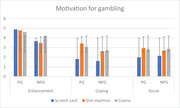

## 1 INTRODUCTION

In the last 20 years, gambling has become more and more widespread in Italy, with an impressive growth in turnover and gamblers (Esposito, 2014; UPB: Ufficio Parlamentare di Bilancio, 2018). A dynamic slowed down by the Covid‐19 emergency (Lugo et al., 2021), but subsequently quickly recovered although with a significant transfer to online games (Agenzia delle Accise, Dogane e Monopoli, 2022). Notwithstanding recreational gambling is often without negative consequences, a minority of gamblers experiences significant harm in relation to their gambling, increasingly recognized as a major public health concern (Abbott, 2020). The prevalence of pathological gambling is estimated to be between 0.1% and 5% worldwide (Calado & Griffiths, 2016; Potenza et al., 2019) and around 2% in Italy (Barbaranelli et al., 2013), while problematic gamblers are far more, among the recreational or occasional gamblers. In the general population, different scales are used to distinguish problematic and nonproblematic gamblers (e.g., the South Oaks Gambling Screen (Lesieur & Blume, 1987); or the Problem Gambling Severity Index (Holtgraves, 2009). The Lie‐Bet tool (E. E. Johnson et al., 1997), a two‐question survey, has proved to be fast and reliable to identify problematic gamblers in several studies on general population (Blanco et al., 2000; Calado et al., 2017; Molinaro et al., 2014). Its sensitivity, specificity, and accuracy to differentiate problem from nonproblem gamblers has been confirmed in several studies, which have shown good convergence with other diagnostic tools, that is, the full DSM‐IV Criteria (Götestam et al, 2004), Problem Gambling Severity Index (Wieczorek et al, 2021), or Canadian Problem Gambling Index (Colasante et al, 2013). Other studies show moderate convergence, with a tendency to overestimate pathological gamblers (i.e., Rossow & Molde, 2006, with SOGS‐RA). A recent meta‐analysis (Dowling et al, 2019) conducted a systematic search from 1990 to 2019 on 20 most accurate brief screening instruments to identify problem and at‐risk gambling: the analysis show that Lie/Bet displayed satisfactory diagnostic accuracy in general population and nongambling clinical contexts to detected problem gamblers (95% of them were accurately identified), but not at‐risk gamblers. Its diagnostic accuracy was the most robust in contests with high risk of bias.

The DSM‐5, after a long debate, considers gambling an addiction and not an impulse control disorder anymore (Rennert et al., 2014). Following this theoretical evolution, several studies and models have begun to consider the use of gambling for emotion regulation (e.g., to enhance mood, social relationship or to escape unpleasant emotions), similarly to substance use and abuse (Flack & Stevens, 2019). In their model about alcohol consumption, Cooper et al. (1995) hypothesized that the declared motivations underling consumption represent phenomenologically distinct behaviors, having both unique antecedents and consequences, which regulate both positive and negative emotions. Stewart and Zack (2008) investigated the motivations of gambling along the lines of the three‐dimensional measure of drinking motives (Cooper et al., 1992; Grande‐Gosende et al., 2019; Lambe et al., 2015): coping (internal, negative reinforcement, i.e., to reduce or avoid negative emotions); enhancement (internal, positive reinforcement, i.e., to increase positive emotions); and social (external, positive reinforcement motives, i.e., to increase social affiliation). This model points out how internal motivations (in particular, Coping) predicted gambling problems, suggesting an association between emotion‐regulation motives and pathological gambling rather than recreational.

Gambling includes different games. Some require skills (e.g., card games), while many others involve pure luck (e.g., lottery). Few studies have investigated how specific motivational dimensions drive the use of different games. Griffiths (1991) points out that adolescents played slot‐machines primarily for excitement and socialization. Clarke (2005) underlines that slot‐machine players gamble for stimulation, tension release, and feelings of importance in the eyes of others. Fang and Mowen (2009) claim that chance games (like slot‐machine) and skilled games (like cards) have divergent motives: the first excitement, escape, and to counteract low self‐esteem, the second social interaction and enhancement. Sundqvist et al. (2016), on the other hand, did not report any differences in different types of gambling (slot, sports betting, casino, lotteries). Flack and Stevens (2019) found that risk gamblers held more favorable views on the emotion focused expected outcomes of gambling compared to no‐risk gamblers and confirm motivational differences between games (escape and excitement in slot‐machine; excitement and social in betting, etc.).

In the light of these considerations, the aim of this study is to observe the relations between gambling motivations, emotions felt while gambling, and types of gambling in an Italian context (Valle d'Aosta, where there is one of the few casinos in the national territory) in a nonclinical population but trying to identify potentially problematic gamblers and their underlying motivations. In particular, we considered three games: slot‐machine (in public premises like cafès pubs, saloons, etc.), casino, and scratch cards, which is a popular activity, similar to lottery but more widespread for their continuous nature, rapid event frequency, and low price (Griffiths, 2002).

## 2 MATERIALS AND METHODS

### 2.1 Participants

A total of 425 gamblers (45.7% F) completed three forms of the questionnaire (one for every type of games) contacted in three different contexts in different town or villages (scratch card vendors *N* = 160, 56.9% F; slot‐machine venues, *N* = 129, 48.8% F; casino *N* = 136, 55.9% F). Mean age was 46.5 (SD = 18.2; range 16–99). The study was conducted in accordance with the Declaration of Helsinki and participants expressed informed consent. According to the Italian Association of Psychology, the approval of and *Ethical committee was not required with adult participants*.

### 2.2 Measures

*The Lie‐Bet two‐question tool* (Johnson et al., 1988) consistently differentiates between pathological gambling and nonproblem‐gambling (Have you ever felt the need to bet more and more money? Have you ever had to lie to people important to you about how much you gambled? If Yes to one or both questions, participants are classified as potentially pathological gamblers (*N* = 162, 38.1%) otherwise not (*N* = 263, 61.9%). At the chi‐square test, no gender difference emerged in the distribution (χ^2^(1,425) = .876, *p* = .349).

*Motivation for gambling* was measured with the Gambling Motives Questionnaire (GMQ; Stewart & Zack, 2008). Participants report the frequency of each of 15 motives on a 7‐point scale from 1 (never) to 7 (always). An exploratory principal components analysis confirmed the three intercorrelated factors found by the authors, each with five times. In the current study sample, reliability confidents for the three subscales were similar to the values reported in previous contributions, with good internal consistency (enhancement (ENH; α = .82, 5 items), coping (COP; α = .85, 5 items), and social (SOC; α = .78, 5 items) motives.

*Emotions* were measured with a list of 8 positive (e.g., joy, curiosity, hope) and 10 negative emotions (sadness, anxiety, fear). Participants were asked to rate the intensity of each emotion usually felt when playing SLOT‐MACHINES/SCRATCH CARD/tables at the CASINO, on a 7‐point scale from 1 (not at all) to 7 (very much). Two global indexes were obtained averaging all the positive (α = .86) and negative emotions (α = .91).

At the end, participants were asked to fill the sociodemographic form.

## 3 RESULTS AND DISCUSSION

Table 1 presents descriptive data and the correlations among motives and emotions. The more relevant motive is Enhancement ad positive emotions are felt more intensively than negative ones.

**TABLE 1:** Correlations among motives and emotions.

	2	3	4	5	*M*	SD
1. ENH	.47	.47	.69	.30	4.13	2.01
2. COP		.62	.39	.56	2.47	1.68
3. SOC			.41	.41	2.56	1.45
4. PE				.35	4.05	1.81
5. NE				–	1.94	1.23

*Note*. All correlations are significant at *p* < .001.

ENH, enhancement; COP, coping; SOC, social; PE, positive emotion; NE, negative emotions.

The three subgroups of gamblers were compared on the three subscales of motives, controlling for gender and pathological/nonpathological status, with ANOVAS 3 × 2 × 2 with three between factors (Type of Game × Gender × Pathology).

**FIGURE 1:**
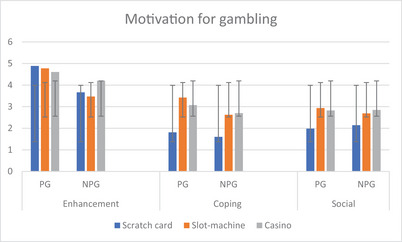
One‐way and post hoc comparison between motives in type of games and pathological/nonpathological gamblers. Error bars represent standard deviations.

For the ENH motive, Type of Game did not reach significance whereas the main effects of Gender (*F*_(1,422)_ = 4.8, *p* = .029, η^2^_p_ = .011) and Pathology (*F*_(1,422)_ = 20.6, *p* = .001, η^2^_p_ = .048) emerged, being more relevant for men (4.35 vs. 3.94) and for pathological gamblers (4.73 vs. 3.76). For the COP motive, Type of Game (*F*_(2,422)_ = 34.7, *p* = .001, η^2^_p_ = .14) and Pathology (*F*_(1,422)_ = 8.6, *p* = .004, η^2^_p_ = .020) were both significant, more relevant for pathological gamblers (*M* = 2.98 vs. 2.16). For SOC motive, the main effect of Type of Game was the only significant effect (*F*_(1.422)_ = 12.3, *p* = .001, η^2^_p_ = .057), partially confirming previous evidence. In Figure 1, the mean values and significant differences at the one‐way post hoc comparisons between the three motives in the three subgroups of gamblers are presented.

For the ENH motives, no subgroups are significantly different at *p* < .05. For both COP and SOC motives, the score is significantly higher for slot‐machine and casino gamblers compared to scratch card gamblers.

**FIGURE 2:**
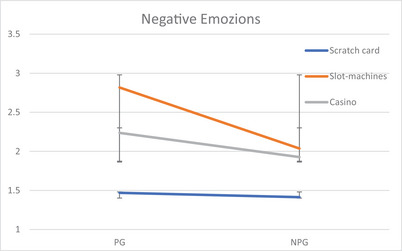
Significant interactions Gender × Pathology and Type of Game × Pathology for the negative emotions felt gambling. Error bars represent standard deviations.

Similar analyses were conducted on the two indexes of positive and negative emotions. No significant differences emerged for the PE with the exception of the main effect of Pathology (*F*_(1,422)_ = 5.8, *p* = .017, η^2^_p_ = .013, 4.3 vs. 3.9, more intense for PG), whereas for the NE Type of Game is significant (*F*_(2,422)_ = 27.9, *p* = .001, η^2^_p_ = .12): NE are more intensively felt by slot‐machine gamblers (*M* = 2.4), followed by casino (*M* = 2.08) and lastly by scratch card (*M* = 1.42) gamblers. Significant differences emerged also for Pathology (*F*_(1,422)_ = 11.4, *p* = .001, η^2^_p_ = .027); comparing PG and NPG, the former felt more intense more intense negative emotions (*M* = 2.33 vs. 1.70). In addition, these effects are qualified by two two‐way interactions: Gender by Pathology (*F*_(1,422)_ = 4.1, *p* = .045, η^2^_p_ = .010) and Type of Game by Pathology (*F*_(2,422)_ = 2.2, *p* = .039, η^2^_p_ = .016). As shown in Figure 2, pathological male gamblers feel more intense negative emotions gambling compared to women; in NPG, the Type of Game does not modify their intensity whereas the negative emotions are more intense in the pathological slot‐machine gamblers.

## 4 CONCLUSIONS

The main aim of the present study was the comparison of motivations and emotions felt while gambling in subgroups of gamblers contacted in different context with different types of games, that is scratch, slot‐machine, and casino gamblers. According to most of the literature (Clarke, 2005; Fang & Mowen, 2009; Flack & Stevens, 2019), different games seem connected to different motivations and emotions. Our findings highlight that the scratch gamblers show less Coping and Social Motivation and experience less Negative Emotion, while Slot and Casino gamblers have more Coping and Social Motivation and feel more Negative Emotions. However, the motivation and emotion with the highest scores (most intensely experienced by the gamblers), the Enhancement Motivation and the Positive Emotions, do not show differences between the different types of games (in line with Sundqvist et al., 2016).

This result might indicate the tendency of gamblers to strive for an increase in Positive Emotions (ENH) regardless of the type of game. In addition, the analysis of correlations between pathological gamblers (PPG) and gender showed that ENH is a more relevant motivation for men (4.35 vs. 3.94) and for PPGs (4.73 vs. 3.76). As for the association of Positive and Negative Emotions (PEs and NEs) and type of game, results revealed that NEs were perceived more intensely by slot‐machine gamblers (*M* = 2.4), followed by casino gamblers (*M* = 2.08), and finally by scratch gamblers (*M* = 1.42). PEs were instead more strongly associated with pathology (*F*_(1,422)_ = 5.8, *p* = .017, η^2^_p_ = .013, 4.3 vs. 3.9) and not with the specific type of game, suggesting that PPs are generally more intense for PPGs, regardless of the type of player.

These results offer a more complete picture if we also consider the differences between pathological and nonpathological gamblers. Pathological gamblers, as classified on the Lie‐Bet questions, represent the 38.1% of our nonclinical samples and report higher Emotions (both Positive and Negative) and internal motives (Enhancement and Coping) than nonpathological gamblers. The pathological gamblers have a more intense and internal connection with the game. In sum, our findings support the theoretical hypothesis of Stewart and Zack (2008): gambling can be a tool for regulating emotions, as for the motivational model on alcohol use and abuse proposed by Cooper et al. (1995). In general, gambling aimed at enhancing positive emotions, while games such as slot‐machines and casino are also aimed at managing negative emotions (Forrest, 2015; Monaci et al., 2005).

This study has several limitations: in particular, the limited number of participants, modest statistical effects, the focus on emotions and the three motivations of the Stewart and Zack's model (2008). In the future, it would be interesting, in addition to increasing the number of participants and games, to investigate other reasons, for example the financial objective (Dechant, 2014; Schellenberg et al., 2016; Tabri , 2022). Additionally, actual gambling behavior factors (gambling time, money spent) and other demographic information should be included in future studies.

## AUTHOR CONTRIBUTIONS

**L. Ferro**: Investigation; conceptualization; writing—review and editing. **M. G. Monaci**: Writing—review and editing; data curation; methodology. **L. Scacchi**: Conceptualization; methodology; project administration; supervision; writing—original draft.

## CONFLICT OF INTEREST STATEMENT

The authors declare that there is no conflict of interest regarding the publication of this paper.

## FUNDING INFORMATION

No financial support was received for this study. Special thanks to University of Valle d'Aosta and Saint‐Vincent Casino for indirectly supporting this project.

### PEER REVIEW

The peer review history for this article is available at https://publons.com/publon/10.1002/brb3.3416.

## Data Availability

The data used to support the findings of this study are available from the corresponding author upon request.
